# The Voxelwise Encoding Model framework: A tutorial introduction to fitting encoding models to fMRI data

**DOI:** 10.1162/imag_a_00575

**Published:** 2025-05-09

**Authors:** Tom Dupré la Tour, Matteo Visconti di Oleggio Castello, Jack L. Gallant

**Affiliations:** Helen Wills Neuroscience Institute, University of California, Berkeley, CA, United States; Department of Neuroscience, University of California, Berkeley, CA, United States

**Keywords:** voxelwise encoding models, fMRI data analysis, functional brain mapping, predictive modeling, ridge regression, naturalistic stimuli

## Abstract

The Voxelwise Encoding Model framework (VEM) is a powerful approach for functional brain mapping. In the VEM framework, features are extracted from the stimulus (or task) and used in an encoding model to predict brain activity. If the encoding model is able to predict brain activity in some part of the brain, then one may conclude that some information represented in the features is also encoded in the brain. In VEM, a separate encoding model is fitted on each spatial sample (i.e., each voxel). VEM has many benefits compared to other methods for analyzing and modeling neuroimaging data. Most importantly, VEM can use large numbers of features simultaneously, which enables the analysis of complex naturalistic stimuli and tasks. Therefore, VEM can produce high-dimensional functional maps that reflect the selectivity of each voxel to large numbers of features. Moreover, because model performance is estimated on a separate test dataset not used during fitting, VEM minimizes overfitting and inflated Type I error confounds that plague other approaches, and the results of VEM generalize to new subjects and new stimuli. Despite these benefits, VEM is still not widely used in neuroimaging, partly because no tutorials on this method are available currently. To demystify the VEM framework and ease its dissemination, this paper presents a series of hands-on tutorials accessible to novice practitioners. The VEM tutorials are based on free open-source tools and public datasets, and reproduce the analysis presented in previously published work.

## 1 Introduction

The Voxelwise Encoding Model (VEM) framework is a powerful approach for functional brain mapping. The VEM framework builds upon encoding models to map brain representations from functional magnetic resonance imaging (fMRI) recordings. (See**Section 2**for a brief overview of the VEM framework.) Over the last two decades, the VEM framework has been used to map brain representations generated by visual images (Agrawal et al., 2014;Dumoulin & Wandell, 2008;Eickenberg et al., 2017;Güçlü & van Gerven, 2015;Hansen et al., 2004;Kay, Naselaris, et al., 2008;Konkle & Alvarez, 2022;Naselaris et al., 2009;Stansbury et al., 2013;St-Yves & Naselaris, 2018;Thirion et al., 2006), movies (Çukur et al., 2013;Dupré la Tour et al., 2021;Eickenberg et al., 2017;Huth et al., 2012;Khosla et al., 2021;Lescroart & Gallant, 2019;Nishimoto et al., 2011;Popham et al., 2021;Wen et al., 2018), music and sounds (Kell et al., 2018;Schönwiesner & Zatorre, 2009), semantic concepts (Deniz et al., 2023;Mitchell et al., 2008;Wehbe et al., 2014), and narrative language (Caucheteux & King, 2022;de Heer et al., 2017;Deniz et al., 2019;Huth et al., 2016;Jain & Huth, 2018;Jain et al., 2020;Toneva & Wehbe, 2019).

The VEM framework provides several critical improvements over other fMRI data analysis methods. First, most procedures for analyzing fMRI data can only accommodate a small number of different conditions (Friston et al., 1994;Penny et al., 2011;Poline & Brett, 2012). In contrast, VEM can efficiently analyze many different stimulus and task features simultaneously. This enables the analysis of complex naturalistic stimuli and tasks, as advocated by many (Dubois & Adolphs, 2016;Hamilton & Huth, 2020;Hasson et al., 2010;Matusz et al., 2019;Wu et al., 2006).

Second, experiments in many areas of neuroscience suffer from a lack of reproducibility (Open Science Collaboration, 2015) and an inflated rate of false positives (Type I error;Bennett et al., 2009;Button et al., 2013;Cremers et al., 2017). This is caused by an over-reliance on null hypothesis testing, an underappreciation of the importance of prediction accuracy and generalization in model selection, and a failure to control overfitting (Wu et al., 2006;Yarkoni, 2020;Yarkoni & Westfall, 2017). In contrast, VEM is a predictive modeling framework that evaluates model performance on a separate test dataset. Because the test dataset contains brain responses to stimuli that were not used during model estimation, voxelwise encoding models are tested directly for their ability to generalize to new stimuli. Because these models are typically estimated and evaluated in individual participants, each participant has their own training and test data and provides a complete replication of all hypothesis tests. These methodological choices ensure that VEM is not prone to overfitting and inflated Type I error, and that the results generalize to new stimuli and participants.

Third, most studies project data from individuals into a standardized template space and then average over the group, ignoring the substantial individual differences that have been observed in both anatomical and functional fMRI data (Dubois & Adolphs, 2016;Gordon et al., 2017;Tomassini et al., 2011;Valizadeh et al., 2018). In contrast, to maintain maximal spatial and functional resolution, VEM typically performs all analyses in each participant’s native brain space without unnecessary spatial smoothing or averaging.

Fourth, most methods produce simple statistical brain maps (e.g., SPM,Friston et al., 1994; MVPA,Haxby et al., 2014; RSA,Kriegeskorte et al., 2008). In contrast, VEM produces high-dimensional functional maps that reflect the selectivity of each voxel to thousands of stimulus and task features.

Despite these critical improvements, the use of VEM in neuroimaging is still relatively limited. This is likely because many in the fMRI community have not received training in modern methods of data science, and the lack of detailed instructions about how to fit encoding models to fMRI data (but seeAshby, 2019, Chapter 15;Naselaris et al., 2011; andhttps://github.com/HuthLab/speechmodeltutorial). Several recent efforts have sought to provide educational materials to support other neuroimaging methods, such as BrainIAK (Kumar et al., 2020,^2021^), Neurohackademy (https://neurohackademy.org/course_type/lectures/), Neuromatch (https://academy.neuromatch.io), Dartbrains (Chang, Huckins, et al., 2020) (https://dartbrains.org), or Naturalistic-Data (Chang, Manning, et al., 2020) (https://naturalistic-data.org). These educational projects cover a wide variety of topics in neuroimaging, and complement other existing resources from well-documented toolboxes such as*PyMVPA*(Hanke et al., 2009) and*nilearn*(Abraham et al., 2014). However, these existing projects do not provide a sufficient foundation for new users to use VEM to implement and test encoding models.

To demystify VEM and ease its dissemination, we have created a series of hands-on tutorials that should be accessible to novice practitioners. These tutorials use free open-source tools that have been developed by our lab and by the scientific Python community (seeSection 3). The tutorials are based on a public dataset (Huth et al., 2022) and they reproduce some of the analyses presented in published work from our lab (Huth et al., 2022,^2012^;Nishimoto et al., 2011;Nunez-Elizalde et al., 2019). These tutorials were presented at the 2021 conference on Cognitive Computational Neuroscience (CCN;https://2021.ccneuro.org/event0198.html?e=3; video recording available athttps://www.youtube.com/watch?v=jobQmEJpbhY).

This document provides a guide to the online tutorials. The second section of this guide presents a brief overview of the VEM framework. The third section describes the technical implementation of the VEM tutorials, listing the different tools used therein and the associated design decisions. The fourth section provides a brief overview of the VEM tutorials content. The fifth section highlights some of the key analyses performed in the tutorials.

## 2 The Voxelwise Encoding Model Framework

A fundamental problem in neuroscience is to identify the information represented in different brain areas. In the VEM framework, this problem is solved using encoding models. An encoding model describes how various features of the stimulus (or task) predict the activity in some part of the brain (Wu et al., 2006). Using VEM to fit an encoding model to blood oxygen level-dependent signals (BOLD) recorded by fMRI involves several steps. First, brain activity is recorded while subjects perceive a stimulus or perform a task. Then, a set of features (that together constitute one or more*feature spaces*) is extracted from the stimulus or task at each point in time. For example, a video might be represented in terms of the amount of motion in each part of the screen (Nishimoto et al., 2011), or in terms of semantic categories of the objects present in the scene (Huth et al., 2012). Each feature space corresponds to a different representation of the stimulus- or task-related information. The VEM framework aims to identify if each feature space is encoded in brain activity. Each feature space, thus, corresponds to a hypothesis about the stimulus- or task-related information that might be represented in some part of the brain. To test this hypothesis for some specific feature space, a regression model is trained to predict brain activity from that feature space. The resulting regression model is called an*encoding model*. If the encoding model predicts brain activity significantly in some part of the brain, then one may conclude that some information represented in the feature space is also represented in brain activity. To maximize spatial resolution, in VEM a separate encoding model is fit on each spatial sample in fMRI recordings (on each voxel), leading to*voxelwise encoding models*.

Before fitting a voxelwise encoding model, it is sometimes possible to estimate an upper bound of the model prediction accuracy in each voxel. In VEM, this upper bound is called the noise ceiling, and it is related to a quantity called the explainable variance (Hsu et al., 2004;Sahani & Linden, 2002;Schoppe et al., 2016). The explainable variance quantifies the fraction of the variance in the data that is consistent across repetitions of the same stimulus. Because an encoding model makes the same predictions across repetitions of the same stimulus, the explainable variance is the fraction of the variance in the data that can be explained by the model.

To estimate the prediction accuracy of an encoding model, metrics like the coefficient of determination (*R^2^*) or the correlation coefficient (*r*) are used to quantify the similarity between the model prediction and the recorded brain activity. However, higher-dimensional encoding models are more likely to overfit to the training data. Overfitting causes inflated prediction accuracy on the training set and poor prediction accuracy on new data. To minimize the chances of overfitting and to obtain a fair estimate of prediction accuracy, the comparison between model predictions and brain responses must be performed on a separate test data set that was not used during model training. The ability to evaluate a model on a separate test data set is a major strength of the VEM framework. It provides a principled way to build complex models while limiting the amount of overfitting. To further reduce overfitting, the encoding model is regularized. In VEM, regularization is obtained by ridge regression (Hoerl & Kennard, 1970), a common and powerful regularized regression method.

To take into account the temporal delay between the stimulus and the corresponding BOLD response (i.e., the hemodynamic response), the features are duplicated multiple times using different temporal delays (Dale, 1999). The regression then estimates a separate weight for each feature and for each delay. In this way, the regression builds for each feature the best combination of temporal delays to predict brain activity. This combination of temporal delays is sometimes called a finite impulse response (FIR) filter (Ashby, 2019;Goutte et al., 2000;Kay, David, et al., 2008). By estimating a separate FIR filter per feature and per voxel, VEM does not assume a unique hemodynamic response function.

After fitting the regression model, the model prediction accuracy is projected on the cortical surface for visualization. Our lab created the*pycortex*visualization software specifically for this purpose (Gao et al., 2015). These prediction-accuracy maps reveal how information present in the feature space is represented across the entire cortical sheet. (Note that VEM can also be applied to other brain structures, such as the cerebellum (LeBel et al., 2021) and the hippocampus. However, those structures are more difficult to visualize.)

In an encoding model, all features are not equally useful to predict brain activity. To interpret which features are the most useful to the model, VEM uses the fit regression weights as a measure of relative importance of each feature. A feature with a large absolute regression weight has a large impact on the predictions, whereas a feature with a regression weight close to zero has a small impact on the predictions. Overall, the regression weight vector describes the*voxel tuning*, that is, the feature combination that would maximally drive the measured activity in a voxel. To visualize these high-dimensional feature tunings over all voxels, feature tunings are projected on fewer dimensions with principal component analysis, and the first few principal components are visualized over the cortical surface (Huth et al., 2012,^2016^). These tuning maps reflect the selectivity of each voxel to thousands of stimulus and task features.

In VEM, comparing the prediction accuracy of different feature spaces within a single data set amounts to comparing competing hypotheses about brain representations. In each voxel, the best-predicting feature space corresponds to the best hypothesis about the information represented in that voxel. However, many voxels represent multiple feature spaces simultaneously. To take this possibility into account, in VEM a joint encoding model is fit on multiple feature spaces simultaneously. The joint model automatically combines the information from all feature spaces to maximize the joint prediction accuracy.

Because different feature spaces used in a joint model might require different regularization levels, VEM uses an extended form of ridge regression that provides a separate regularization parameter for each feature space. This extension is called*banded ridge regression*(Nunez-Elizalde et al., 2019). Banded ridge regression also contains an implicit feature-space selection mechanism that tends to ignore feature spaces that are non-predictive or redundant (Dupré la Tour et al., 2022). This feature-space selection mechanism helps to disentangle correlated feature spaces and it improves generalization to new data.

To interpret the joint model, VEM implements a variance decomposition method that quantifies the separate contributions of each feature space. Variance decomposition methods include variance partitioning (Lescroart et al., 2015), the split-correlation measure (St-Yves & Naselaris, 2018), or the product measure (Dupré la Tour et al., 2022). The obtained variance decomposition describes the contribution of each feature space to the joint encoding model predictions.

## 3 Tutorials Design

The VEM tutorials are written in the Python programming language, and therefore benefit from a collection of freely-available open-source tools developed by the scientific Python community. This section lists these tools and describes the associated design decisions.

### 3.1 Basic knowledge required

The VEM tutorials require some (beginner) skills in Python and*numpy*(Harris et al., 2020). For an introduction to Python and*numpy*, we refer the reader to existing resources such as the scientific Python lectures (https://scipy-lectures.org/), or the*numpy*absolute beginner guide (https://numpy.org/devdocs/user/absolute_beginners.html). Additionally, the VEM tutorials use terminology from*scikit-learn*(Pedregosa et al., 2011). For an introduction to*scikit-learn*and its terminology, we refer the reader to the*scikit-learn*getting-started guide (https://scikit-learn.org/stable/getting_started.html) and glossary of common terms (https://scikit-learn.org/stable/glossary.html).

### 3.2 Tutorial format

The VEM tutorials are open source (BSD-3-Clause license) and freely available to the scientific community. They are under continuous development and open to external contributions. To track changes of the codebase over time, the code is version-controlled using*git*and hosted on the GitHub platform (https://github.com/gallantlab/voxelwise_tutorials). To make sure the code remains functional during development, the code and the tutorials are continuously tested using*pytest*and GitHub Actions.

To satisfy different user preferences, the VEM tutorials can be accessed in multiple ways. First, the VEM tutorials can be downloaded and run locally as*jupyter*notebooks (Kluyver et al., 2016). Second, the notebooks with GPU support can be run for free in the cloud using Google Colab (free Google account required). In this case, we concatenated all notebooks into a single notebook for convenience. Third, the VEM tutorials can be explored pre-rendered on a dedicated website (https://gallantlab.org/voxelwise_tutorials) hosted for free by GitHub Pages. The website is built with*jupyter-book*(Executable Books Community, 2020) and provides an easy way to navigate through the available tutorials.

### 3.3 Docker support

To facilitate running the tutorials locally in different computing environments, we provide Dockerfiles to build containers that include all required dependencies. These files are available in the GitHub repository and are based on Neurodocker (https://www.repronim.org/neurodocker/). Users can generate Docker containers with either CPU or GPU support, depending on their available hardware.

### 3.4 Dataset

**Fig. 1.**
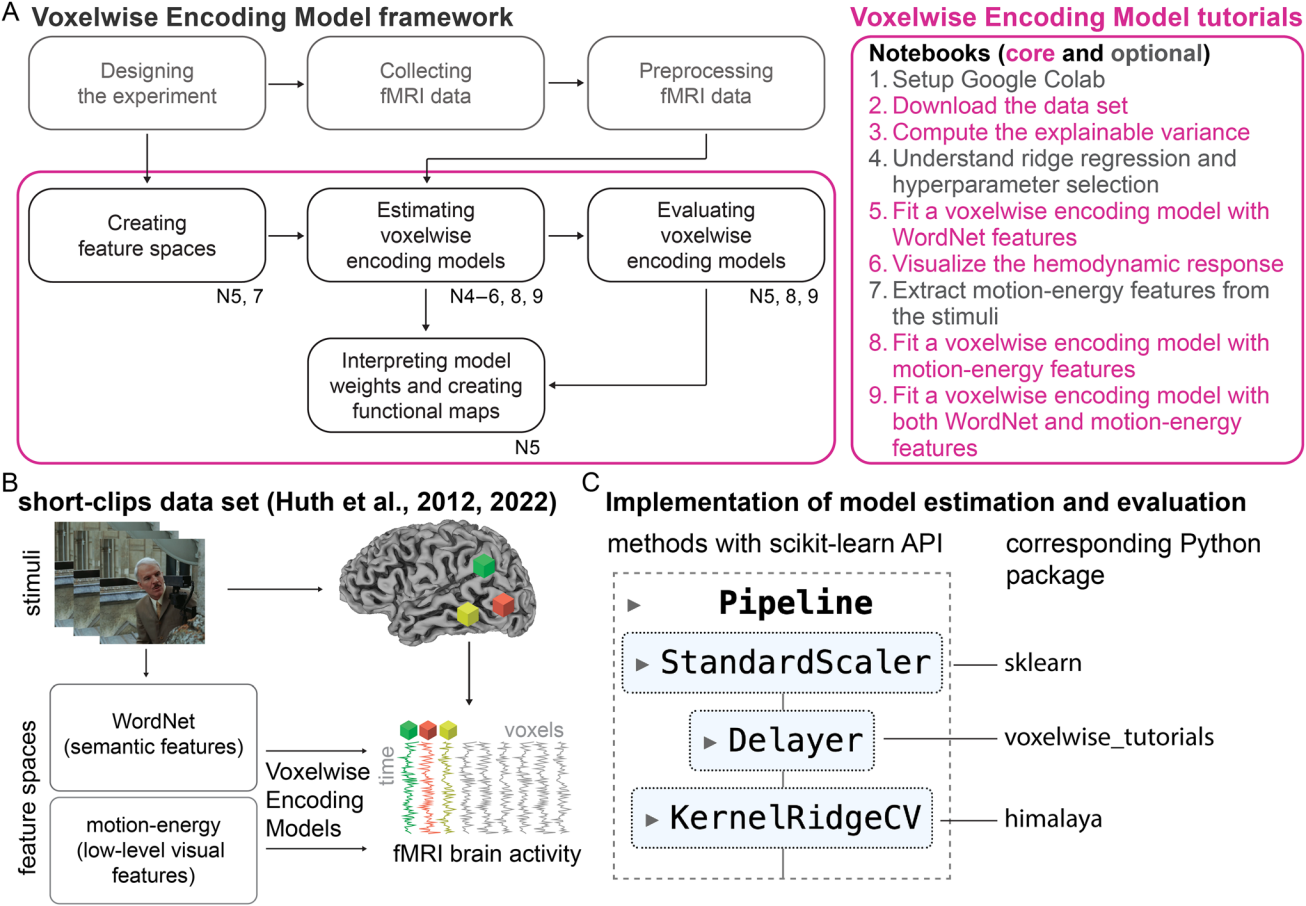
Overview of the Voxelwise Encoding Model tutorials. (A) The Voxelwise Encoding Model framework can be divided into seven interacting components related to data collection, model estimation and evaluation, and model interpretation. The tutorials described here cover the practical implementation of the model estimation, evaluation, and interpretation components, highlighted by the colored box. The labels under each component indicate which notebooks cover that component. The notebooks are divided into six core notebooks (highlighted in pink) and three optional notebooks (in gray). The optional notebooks include a tutorial for setting up Google Colab (useful when teaching these tutorials in a class); a tutorial about ridge regression, cross-validation, and the effect of hyperparameter selection on model prediction; and a tutorial that shows how to use*pymoten*(a Python package developed by our lab;Nunez-Elizalde et al., 2024) to extract motion-energy features from the stimuli provided in the public dataset. (B) The tutorials are based on the public data fromHuth et al. (2012). In that experiment, participants watched a series of short clips without sound while their brain activity was measured with fMRI. The public dataset contains two feature spaces: WordNet, which quantifies the semantic information in the stimuli, and motion-energy, which quantifies low-level visual features. Both feature spaces are used in the tutorials. (C) Model estimation and model evaluation methods are implemented with a fully compatible scikit-learn API. For example, a voxelwise encoding model estimated with regularized regression is implemented as a scikit-learn*Pipeline*consisting of a*StandardScaler*step (a preprocessing scikit-learn step to standardize the features prior to model fitting), a*Delayer*step (a method implemented in the associated*voxelwise_tutorials*Python package to delay the features prior to model fitting), and a*KernelRidgeCV*step (an efficient implementation of kernel ridge regression with cross-validation, implemented in the*himalaya*package developed by our lab).

The “short-clips” dataset used in the VEM tutorials contains BOLD fMRI measurements made while human subjects viewed a set of naturalistic, short movie clips (Huth et al., 2012;Nishimoto et al., 2011) (Fig. 1). The dataset is publicly available on the free GIN platform (https://gin.g-node.org/gallantlab/shortclips;Huth et al. (2022)). For convenience, the dataset is additionally hosted on a premium cloud provider with high bandwidth (Wasabi). To download the dataset, a custom user-friendly data loader is available in the VEM tutorials. By using*git-annex*and*datalad*(Halchenko et al., 2021), the data loader can seamlessly switch between the different data sources depending on their availability. The data are stored using the HDF5 format, which enables direct access to array slices without loading the entire file in memory. The read/write operations are performed using*h5py*.

### 3.5 Regression methods

Voxelwise encoding models are based on regression methods such as ridge regression (Hoerl & Kennard, 1970) and banded ridge regression (Nunez-Elizalde et al., 2019). Because a different model is estimated for every voxel, and because a typical fMRI dataset at 3T contains about 10^5^voxels, fitting voxelwise encoding models can be computationally challenging. To address this challenge, the VEM tutorials use algorithms optimized for large numbers of voxels, implemented in*himalaya*(Dupré la Tour et al., 2022). To further improve computational speed,*himalaya*provides three different computational backends to fit regression models either on CPU (using*numpy*,Harris et al., 2020, and*scipy*,Virtanen et al., 2020) or on GPU (using either*pytorch*,Paszke et al., 2019, or*cupy*,Okuta et al., 2017). The VEM tutorials also use*scikit-learn*(Pedregosa et al., 2011) to define the regression pipeline and the cross-validation scheme.

### 3.6 Additional tools

Beyond the software infrastructure that underlies VEM, the tutorials also provide a custom Python package called*voxelwise_tutorials*. This package contains a collection of helper functions used throughout the VEM tutorials. For example, it includes tools to define the regression pipeline, and generic visualization tools based on*matplotlib*(Hunter, 2007),*networkx*(Hagberg et al., 2008), and*nltk*(Bird & Loper, 2004). To preserve participant privacy, the dataset used in the VEM tutorials does not contain any anatomical information. For this reason, the cortical visualization package*pycortex*(Gao et al., 2015) cannot be used. Instead, the*voxelwise_tutorials*package provides tools to replicate*pycortex*visualization functions using subject-specific*pycortex*mappers that are included with the dataset.

## 4 Tutorials Overview

In the VEM framework, a typical study can be decomposed into seven components: (I) experimental design, (II) data collection, (III) data preprocessing, (IV) feature space extraction, (V) voxelwise encoding model estimation, (VI) model evaluation, and (VII) model interpretation (Fig. 1). These components are described in detail in our comprehensive guide to the VEM framework (Visconti di Oleggio Castello et al., n.d.). Here, we briefly summarize their overarching goals.

The first three components of the VEM framework (experimental design, data collection, and data preprocessing) build the foundations for a successful cognitive neuroscience experiment. These components address the need to choose an appropriate experimental design that reliably activates the sensory, cognitive, or motor representations of interest; to collect fMRI data with high signal-to-noise (SNR) ratio; and to preprocess the collected data to increase SNR and prepare the data for model estimation.

The flexibility of the VEM framework allows researchers to choose any experimental design, from classic controlled tasks to naturalistic experiments such as the movie-watching experiment used in these tutorials. Because voxelwise encoding models are generally estimated and evaluated in individual participants, the preprocessing pipeline used in VEM typically includes only a minimal set of spatial preprocessing steps (motion correction, distortion correction, and co-registration to the participant’s reference volume) and temporal preprocessing steps (detrending, denoising, and time-series normalization). These preprocessing steps can be implemented either in custom preprocessing pipelines (e.g., with*nipype*,Esteban et al., 2020;Gorgolewski et al., 2011) or by using fMRIPrep (Esteban et al., 2019).

The last four components of the VEM framework (feature space extraction, voxelwise encoding model estimation, model evaluation, and model interpretation) address the goal of testing specific hypotheses about brain representations. Testing hypotheses in the VEM framework consist of three steps. First, feature spaces are extracted from the stimulus or task to quantify specific hypotheses. Second, these feature spaces are used to estimate voxelwise encoding models, which are then evaluated on a held-out test dataset. If the encoding model significantly predicts brain activity, then this is taken as support for the hypothesis. Finally, the estimated model weights are interpreted to recover the voxel tuning to specific features in the feature space and to generate functional brain maps.

The present tutorials cover the practical implementation of the last four components of the VEM framework. The VEM tutorials are organized into nine notebooks that are best worked through in order. This section briefly describes the content of each notebook. The next section showcases four notebooks that cover key aspects of the VEM framework related to quality assurance, model estimation and evaluation, and model interpretation.

1. **Setup Google Colab (optional).**This optional notebook describes how to set up the VEM tutorials to run in the cloud using Google Colab. This notebook should be skipped when running the VEM tutorials on a local machine.
2. **Download the data set.**This notebook describes how to download the “short-clips” dataset (Huth et al., 2012,^2022^). This dataset contains BOLD fMRI responses in human subjects viewing a set of natural short movie clips. The dataset contains responses from five subjects, and the VEM analysis can be replicated in each subject independently.
3. **Compute the explainable variance.**This notebook provides a first glance at the data and describes how to compute the explainable variance. The explainable variance quantifies the fraction of the variance in the data that is consistent across repetitions of the same stimulus. This notebook also demonstrates how to plot summary statistics of each voxel onto a subject-specific flattened cortical surface using*pycortex*mappers. This visualization technique is used multiple times in the subsequent notebooks.
4. **Understand ridge regression and hyperparameter selection (optional).**This optional notebook presents ridge regression (Hoerl & Kennard, 1970), a regularized regression method used extensively in VEM. The notebook also describes how to use cross-validation to select the optimal hyperparameter in ridge regression. This notebook is not specific to VEM, and can be skipped by readers who are already familiar with ridge regression and hyperparameter selection.
5. **Fit a voxelwise encoding model with WordNet features.**This notebook shows how to fit a voxelwise encoding model to predict BOLD responses. The encoding model is fit with semantic features extracted from the movie clips, reproducing part of the analysis presented inHuth et al. (2012).
6. **Visualize the hemodynamic response.**This notebook describes how to use finite-impulse response (FIR) filters in voxelwise encoding models to take into account the hemodynamic response in BOLD signals.
7. **Extract motion-energy features from the stimuli (optional).**This optional notebook describes how to use*pymoten*(Nunez-Elizalde et al., 2024) to extract motion-energy features from the stimulus. Motion-energy features are low-level visual features extracted from the movie clips stimuli using a collection of spatio-temporal Gabor filters. This computation can take a few hours, so we have included precomputed motion-energy features in the short-clips dataset.
8. **Fit a voxelwise encoding model with motion-energy features.**This notebook shows how to fit a voxelwise encoding model with different features than in Notebook 5. Here, the encoding model is fit with motion-energy features extracted from the movie clips, reproducing part of the analysis presented inNishimoto et al. (2011). Motion-energy features are low-level visual features extracted in Notebook 7. The voxelwise encoding model is fit using the same method as with semantic features (see Notebook 5). The semantic model and the motion-energy model are then compared over the cortical surface by comparing their prediction accuracies.
9. **Fit a voxelwise encoding model with both WordNet and motion-energy features.**This notebook shows how to fit a voxelwise encoding model with two feature spaces jointly, the semantic and the motion-energy feature spaces.

## 5 Key Tutorials

### 5.1 Notebook 3: Compute the explainable variance

**Fig. 2.**
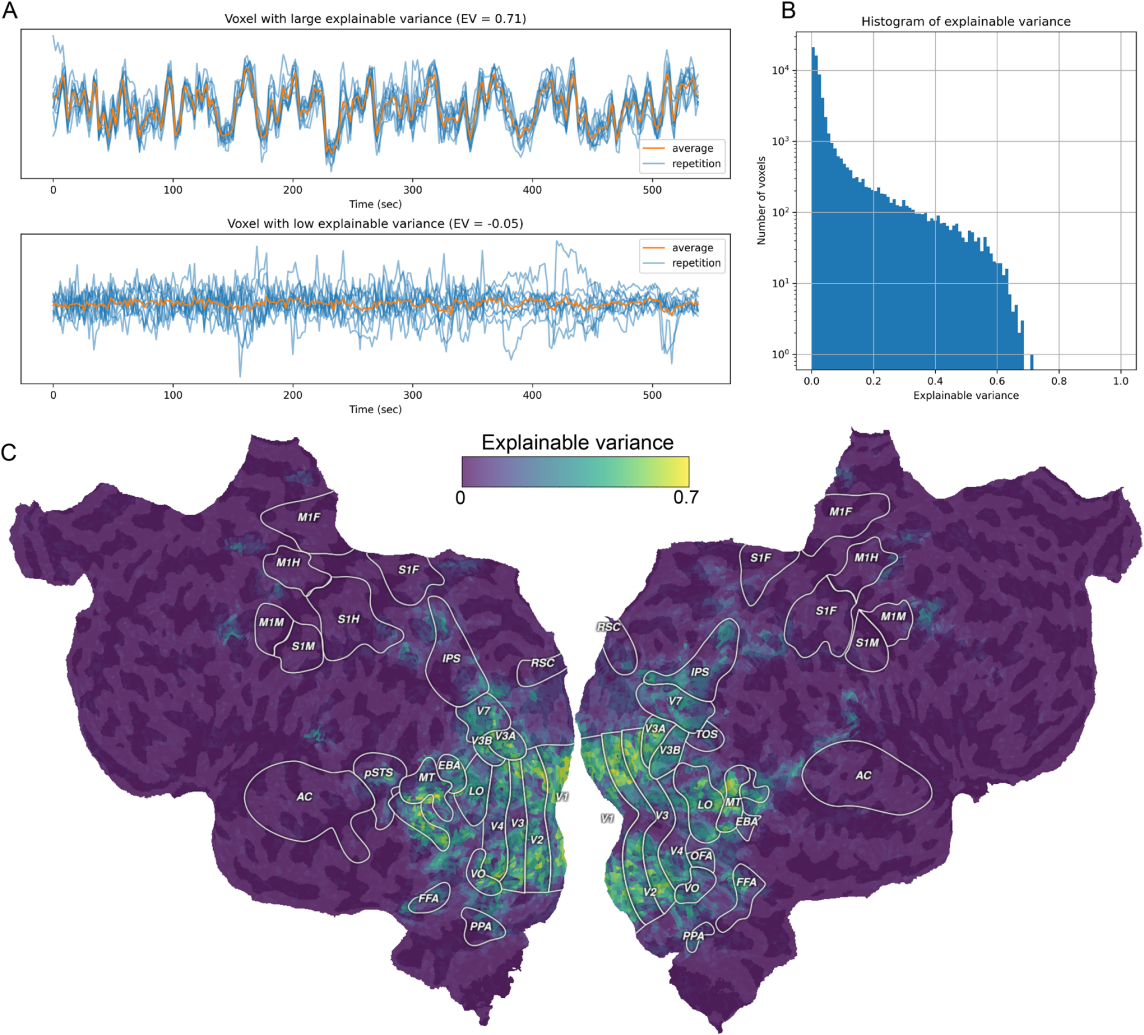
Explainable variance. Explainable variance is proportional to the maximum prediction accuracy that can be achieved by any voxelwise encoding model, given the available test data. For each voxel, explainable variance quantifies the variance in the measured signal that is repeatable. (A) Measured brain responses to 10 repetitions of the same test stimulus for a voxel with high explainable variance (top) and a voxel with low explainable variance (bottom). (B) Histogram of explainable variance across the cortex of one participant. (C) Explainable variance estimates projected onto the cortical surface of one participant. A voxelwise encoding model will be able to predict responses only in voxels with explainable variance greater than zero. Voxels with explainable variance equal or close to zero are either too noisy or not activated reliably by the experiment.

This notebook describes how to compute and interpret explainable variance. Explainable variance is proportional to the maximum prediction accuracy that can be achieved by any voxelwise encoding model, given the available test data (Hsu et al., 2004;Sahani & Linden, 2002;Schoppe et al., 2016). In practice, this is quantified as the fraction of the variance in the data that is consistent across repetitions of the same stimulus. In the VEM framework, computing and visualizing explainable variance is a key quality assurance step, ensuring that the measured data provide enough signal to reliably estimate voxelwise encoding models (Fig. 2).

### 5.2 Notebook 5: Fit a voxelwise encoding model with WordNet features

**Fig. 3.**
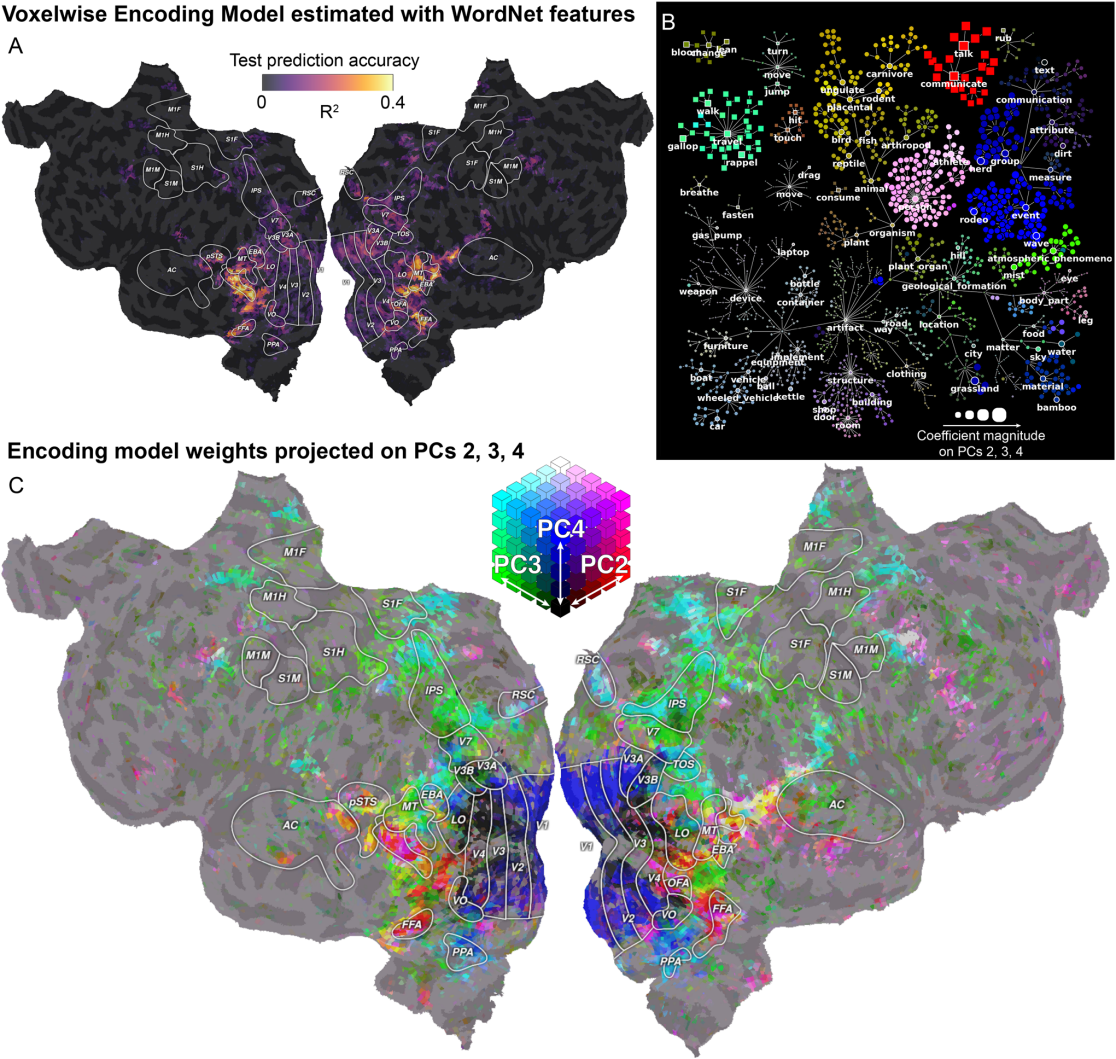
Recovering visual semantic representations during movie watching. The tutorial in Notebook 5 (*Fit a voxelwise encoding model with WordNet features*) shows how to estimate, evaluate, and interpret a voxelwise encoding model fit with the WordNet feature space. This tutorial replicates the analyses of the original publication (Huth et al., 2012) in a single participant. (A) Model prediction accuracy on the held-out test set. The voxelwise encoding model based on semantic WordNet features predicts brain responses in category-specific high-level visual areas, but because low-level and high-level features are correlated, it also predicts responses in motion-sensitive and early visual areas. The tutorial in Notebook 9 (*Fit a voxelwise encoding model with both WordNet and motion-energy features*) will show how to use banded ridge regression to disentangle low-level visual information from semantic information. (B) The estimated model weights are interpreted according to the WordNet labels by first performing principal component analysis (PCA), and then visualizing the model weight PCs on the WordNet graph. Each node in the graph is one of the WordNet labels that were used to tag the stimulus, and the edges of the graph correspond to the relationship*“is a”*(Huth et al., 2012). The first PC is omitted here as it simply distinguishes objects and actions with motion from buildings and static objects. The second, third, and fourth PCs are shown on the WordNet graph by assigning each of the three PCs to one of the RGB channels. The size of each node reflects the magnitude of the PC coefficients. (C) The model weights are projected onto the second, third, and fourth PC to interpret the voxel tuning toward specific visual semantic categories across the cortex. For example, voxels in the fusiform face area (FFA) and in the extrastriate body area (EBA) are colored in red, pink, and yellow, corresponding to communication-related, person-related, and animal-related WordNet labels.

This notebook demonstrates how to fit a voxelwise encoding model to predict brain responses from semantic features. The semantic features included in the dataset consist of WordNet labels (Huth et al., 2012;Miller, 1995) that have been annotated manually for each two-second block of the movie clips. The voxelwise encoding model is fit with ridge regression, and cross-validation is used to select the optimal regularization hyperparameter. Model prediction accuracy is estimated on a separate test set, and projected onto a subject-specific flattened cortical surface. Finally, the regression weights are summarized using principal component analysis. The first few principal components are projected on a flattened cortical surface, and interpreted using graphs that reflect the relations between the semantic features (Fig. 3).

### 5.3 Notebook 6: Visualize the hemodynamic response

**Fig. 4.**
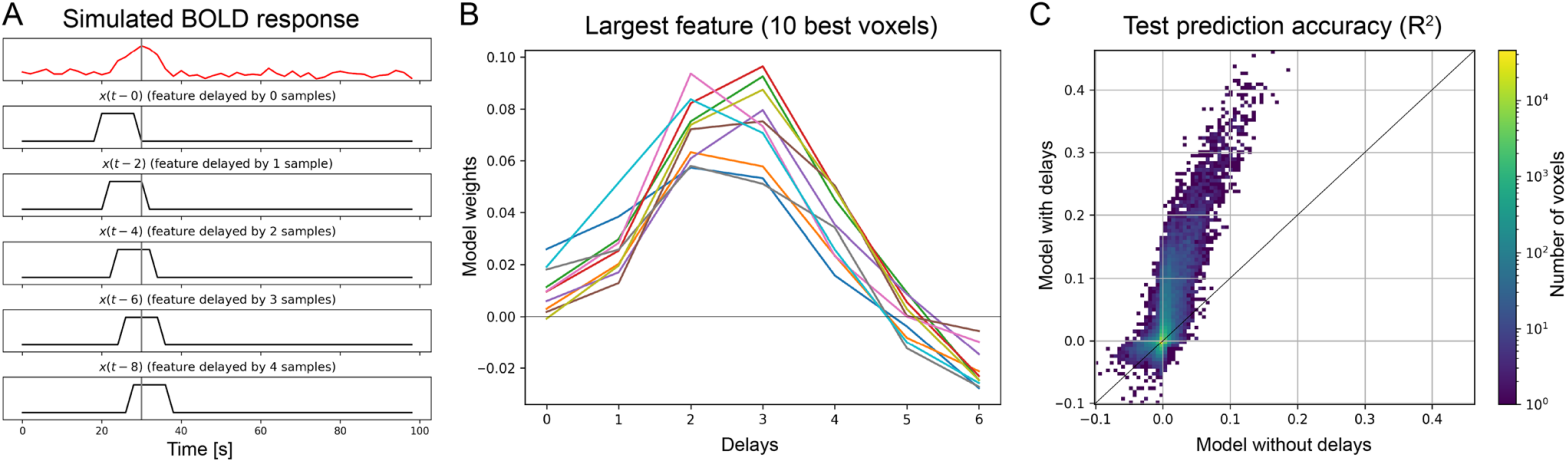
Delaying features to account for the hemodynamic response function. Brain responses measured by fMRI are delayed in time due to the dynamics underlying neurovascular coupling. To account for this delay, in the Voxelwise Encoding Model framework features are delayed and concatenated prior to model fitting. This procedure corresponds to using a finite impulse response (FIR) model to estimate the hemodynamic response function (HRF) in each voxel. (A) Time course of simulated BOLD responses in a voxel (in red) and of a simulated feature (in black). To predict the BOLD response, the corresponding feature is delayed by 0, 1, 2, 3, and 4 samples, corresponding to 0, 2, 4, 6, and 8 s at a TR of 2 s. In this way, the response at time t = 30 s is predicted by a linear combination of the features occurring at times 22, 24, 26, 28, and 30 s. (B) The model weights associated with one feature are displayed for 10 voxels. Using an FIR model allows recovering the shape of the HRF in each voxel. (C) Two-dimensional histogram comparing the test prediction accuracy of a model without delays (x-axis) and a model with delays (y-axis). Using delays improves model prediction accuracy by accounting for the hemodynamic delay in the measured BOLD responses.

This notebook demonstrates how finite-impulse response (FIR) filters are used in voxelwise encoding models to take into account the delayed hemodynamic response in measured BOLD responses. With the FIR method, the features are duplicated multiple times with different temporal delays prior to model fitting. In this way, the encoding model estimates a separate weight per feature, per delay, and per voxel. This approach accounts for the variability of HRFs across voxels and brain areas, and it improves model prediction accuracy (Fig. 4).

### 5.4 Notebook 9: Fit a voxelwise encoding model with both WordNet and motion-energy features

**Fig. 5.**
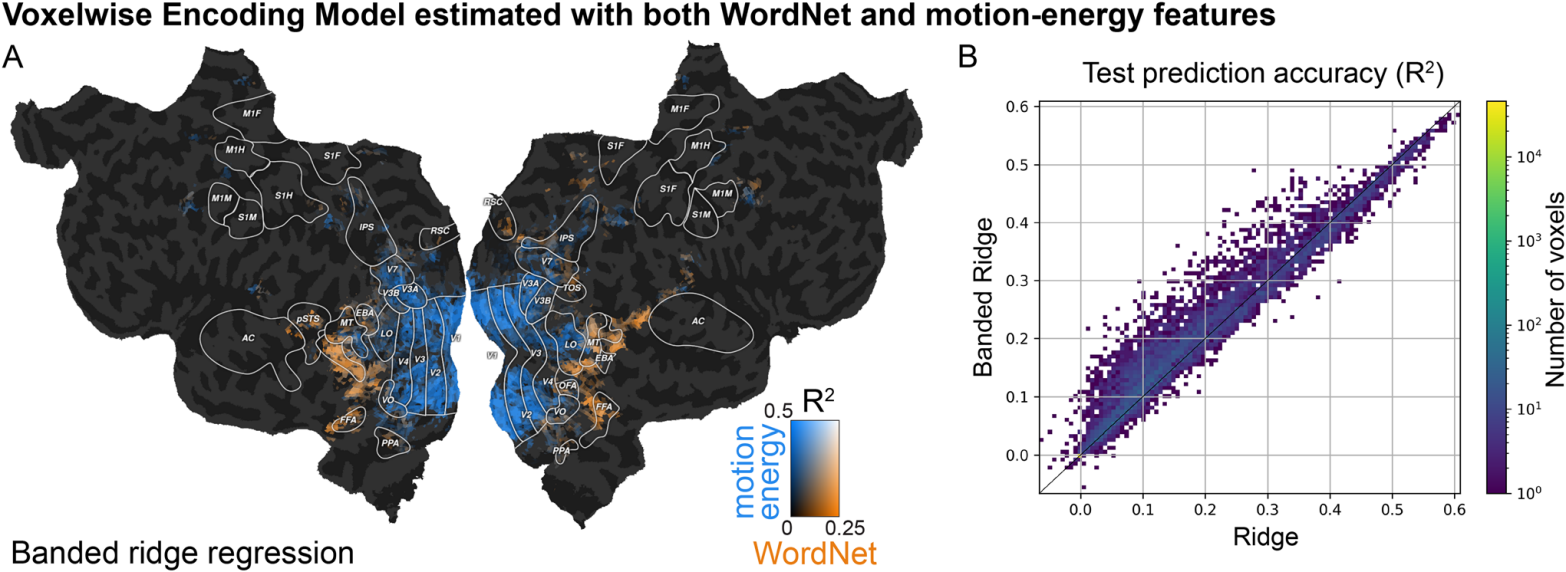
Fitting a voxelwise encoding model with both WordNet and motion-energy features. The tutorial in Notebook 9 (*Fit a voxelwise encoding model with both WordNet and motion-energy features*) shows how to use banded ridge regression to evaluate the contribution of different feature spaces in predicting brain responses. In banded ridge regression, for each voxel two regularization hyperparameters are optimized by performing cross-validation separately for each feature space. (A) Split prediction accuracy on the test set for the motion-energy feature space (blue) and for the WordNet feature space (orange). Compared to the encoding model fit with only the semantic WordNet feature space (Fig. 2), the banded ridge model with both feature spaces disentangles the contribution of low-level visual information on the model prediction from the contribution of semantic information. The WordNet feature space predicts only in category-specific visual areas, while the motion-energy feature space predicts only in early visual areas and motion-sensitive areas. (B) The tutorial also demonstrates the advantage of using banded ridge regression instead of regular ridge regression with more than one feature space. The two-dimensional histogram shows the test prediction accuracy of two encoding models estimated with ridge (x-axis) and banded ridge (y-axis) using both the motion-energy and the WordNet feature spaces. The banded ridge regression model results in more accurate predictions than the ridge regression model.

This notebook demonstrates how to fit a voxelwise encoding model with two feature spaces jointly: the WordNet and the motion-energy feature spaces (see also Notebooks 5, 7, 8). Because the joint model uses two feature spaces, the voxelwise encoding model is fit with banded ridge regression, as presented inNunez-Elizalde et al. (2019). Banded ridge regression adapts the regularization strength for each feature space in each voxel. This improves prediction accuracy by performing implicit feature-space selection (Dupré la Tour et al., 2022). Then, to interpret the contribution of each feature space to the joint model, the joint prediction accuracy is decomposed between both feature spaces (Fig. 5).

## 6 Conclusion

The Voxelwise Encoding Model tutorials provide a solid foundation for creating encoding models with fMRI data. They provide practical examples on how to perform key aspects of the Voxelwise Encoding Model framework, such as performing quality assurance by computing explainable variance, fitting and evaluating voxelwise encoding models with cross-validation and regularized regression, and using PCA to interpret model weights. However, these tutorials are not exhaustive. There are other advanced aspects of Voxelwise Encoding Model that are not yet included in these tutorials. These advanced topics include experimental design, fMRI data acquisition and preprocessing, and using the encoding model framework to analyze measurements made by other means such as neurophysiology or optical imaging (Wu et al., 2006). Because these tutorials are publicly available and open source, they might be augmented in the future to provide more information about these advanced topics.

## Ethics

Data were acquired on human subjects during previous studies (Huth et al., 2012). The experimental protocols were approved by the Committee for the Protection of Human Subjects at University of California, Berkeley. All subjects were healthy, had normal hearing, and had normal or corrected-to-normal vision. Written informed consent was obtained from all subjects.

## Data Availability

The code of the VEM tutorials is available athttps://github.com/gallantlab/voxelwise_tutorials. The short-clips dataset (Huth et al., 2012;Nishimoto et al., 2011) used in the VEM tutorials is available athttps://gin.g-node.org/gallantlab/shortclips(Huth et al., 2022).
